# Continuous care needs in patients with cancer receiving chemotherapy during the recent omicron wave of COVID-19 in Shanghai: A qualitative study

**DOI:** 10.3389/fpsyg.2022.1067238

**Published:** 2023-01-04

**Authors:** Jie Zhang, Caifeng Wang, Lei Huang, Jun Zhang

**Affiliations:** ^1^Department of Nursing, Ruijin Hospital, Shanghai Jiao Tong University School of Medicine, Shanghai, China; ^2^Department of Oncology, Ruijin Hospital, Shanghai Jiao Tong University School of Medicine, Shanghai, China; ^3^School of Nursing, Shanghai Jiaotong University, Shanghai, China; ^4^Medical Center on Aging of Ruijin Hospital, MCARJH, Shanghai Jiao Tong University School of Medicine, Shanghai, China

**Keywords:** COVID-19, continuous care needs, cancer, chemotherapy, qualitative study

## Abstract

**Aims:**

This study aimed to investigate the care needs, to clarify the factors affecting the quality of homecare, and to provide reference for constructing a homecare system for patients with cancer receiving chemotherapy during the recent omicron wave of COVID-19 in Shanghai.

**Methods:**

From March to May 2022 when the omicron wave emerged in Shanghai, 50 consecutive patients who received chemotherapy at Ruijin Hospital, Shanghai Jiao Tong University School of Medicine, were enrolled, and underwent face-to-face or telephone-based semi-structured interviews regarding continuous care needs. Some of their homecare-givers, caring nurses, and physicians were also interviewed. The Colaizzi method was used for data analysis.

**Results:**

Fifty patients, 4 homecare-givers, 4 nurses, and 4 physicians were interviewed. Three themes and six subthemes emerged from analysis of the interviews: The first theme was “Disease management needs,” including needs for knowledge of managing adverse events associated with chemotherapy, and needs for treatment-related information. Patients expressed most concern about not being able to go to the hospital for blood review and disease evaluation in time due to the outbreak. With the COVID-19 pandemic being ongoing, factors such as pandemic panic, inconvenient medical treatment, and worry about hospital cross-infection might reduce disease management for patients with cancer. The second theme was “Medical needs,” including needs for mobile healthcare and needs for medical resources. All interviewees emphasized the importance of mobile healthcare during the COVID-19 pandemic, as access to hospitals was difficult. The third theme was “Spiritual needs,” including demands for psychological counseling and intervention, and needs for spiritual care. Patients and homecare-givers commonly lacked a feeling of security and needed communication, encouragement, and reassurance that medical care could be delivered to them, and patients reported that they very much wanted psychological advice.

**Conclusion:**

For patients with cancer receiving chemotherapy during the COVID-19 pandemic, continuous care is greatly needed. Medical personnel should strengthen the healthcare education for patients and their caregivers during hospitalization, and further improve the patients’ information intake rate through Internet-based digital healthcare methods during homecare, to further meet the information needs of patients after discharge from hospital.

## Introduction

COVID-19 has spread around the world causing a pandemic (Huang et al., 2020a). It can be transmitted rapidly during the asymptomatic period, and is difficult to prevent and control (Aghamirza Moghim Aliabadi et al., 2020; Huang, 2021; Huang et al., 2021a,b,c). Close to the end of February 2022, a new omicron wave of COVID-19 emerged and quickly spread in Shanghai, China (Huang, 2022), and has affected the care of many patients with cancer.

There have been some reports on patients with cancer during the COVID-19 pandemic. A very interesting recent study (Di Lorenzo et al., 2020) investigated 72 patients with metastatic prostate cancer (48 with hormone-sensitive prostate cancer and 24 with castration-resistant prostate cancer) who received hormonal therapy or chemotherapy between March 1 and 27, 2020, in South Italy. Two (8.3%) of the 24 patients with castration-resistant prostate cancer were infected with COVID-19. Both of them were receiving luteinizing hormone-releasing hormone (LHRH) agonist therapy, and one of them was receiving enzalutamide. Urgent intensive care unit (ICU) admission was required for them due to clinical worsening, with blood tests showing severe lymphopenia, anemia, and increased platelet count. Retroviral therapy, antibiotics, heparin, and chloroquine were prescribed at the beginning, and one patient also received tocilizumab as salvage treatment. The patients were discharged from hospital after 3 weeks of hospitalization. Both patients suffered from an aggressive COVID-19 course due to concomitant comorbidities. This study suggested that it could be useful to investigate whether hormonal therapy, especially for patients with advanced prostate cancer, acts as a protective or risk factor during COVID-19. COVID-19 infection in patients with prostate cancer is a complex scenario with multiple facets (Sciarra et al., 2020; Crocetto et al., 2021). A cohort study (Schmidt et al., 2021) suggested that androgen deprivation therapy use was not associated with decreased mortality from SARS-CoV-2 infection in patients with prostate cancer and COVID-19.

A recent article (Esposito et al., 2020) provides very useful and specific control strategies to manage patients during the COVID-19 pandemic which is a rapidly evolving situation and which may delay treatment schedule and disease management (Sciarra et al., 2020; Ferro et al., 2021; Serretta et al., 2022), while preserving the safety of the medical team. COVID-19 has dramatically impacted the activities of many medical staff, especially pertaining to robot-assisted surgery and minimally invasive surgery (MIS). For pediatric surgeons and urologists, it is strongly suggested that surgery should only be performed for pediatric patients with emergent/urgent and oncological indications until resolution of the COVID-19 pandemic. Robotics and MIS may be safely performed in such selected children by adopting specific technical precautions such as prevention of aerosol dispersion using filters/suction or adapted systems and appropriate use of electrocautery and other sealing devices for reduction of surgical smoke. Another recommended key point to cope with the pandemic is that all hospitals should provide healthcare professionals with adequate individual protections and perform universal screening in all patients undergoing surgery (Esposito et al., 2020).

Cancer is a strong risk factor for severe COVID-19 disease (Salomé and Horowitz, 2021), and COVID-19 caused significant morbidity and mortality as well as repeated hospitalizations among cancer patients (Busetto et al., 2020; Manzano et al., 2022). A study (Kim et al., 2022) of 271,639 COVID-19 patients with 18,460 having at least one cancer diagnosis showed that patients with cancer had higher risks for 1-month mortality and hospitalization after SARS-CoV-2 infection compared to those without cancer, and that patients with a cancer diagnosis within 1 year and those receiving active treatment were more vulnerable to worse COVID-19 outcomes. A multicenter study (Plais et al., 2022) further showed that cancer patients who required ICU admission for SARS-CoV-2 infection had an increased mortality rate, with hematological malignancies associated with a higher risk compared to solid cancers. Mechanistically, a COVID-19-dependent pro-inflammatory profile and immune suppression may promote the optimal microenvironment for tumor genesis, progression, metastasis, and recurrence, and immune evasion of malignant cells (du Plessis et al., 2022). Patients with cancer and COVID-19 might also have an elevated thrombosis risk (Li et al., 2021; Thachil et al., 2021).

Further studies investigated management of patients with cancer and COVID-19. Notably, while viral and fungal coinfections were reported to be infrequent among cancer patients with COVID-19, fungal coinfection was associated with a very high mortality rate; guided by clinical and laboratory parameters, early empiric antimicrobial therapy may improve clinical outcomes for cancer patients with COVID-19 (Satyanarayana et al., 2022). The Dutch Oncology COVID-19 Consortium study (de Joode et al., 2022) did not show a negative impact of anticancer therapies on COVID-19 outcomes. Another study (Jee et al., 2020) also showed that recent cytotoxic chemotherapy was not associated with adverse COVID-19 outcomes, but that patients with active hematologic or lung malignancies, peri-COVID-19 lymphopenia, or baseline neutropenia had worse COVID-19 outcomes. However, a multicenter cohort study (Yang et al., 2020) on patients with cancer and COVID-19 in Hubei, China showed that receipt of chemotherapy within 4 weeks before COVID-19 symptom onset was associated with a higher case-fatality rate. Another cohort study (Bakouny et al., 2022) further revealed that in patients with cancer and COVID-19, administration of systemic anticancer therapies, especially immunotherapy, in the context of baseline immunosuppression was associated with severe clinical outcomes and the development of cytokine storm (Turnquist et al., 2020). Preexisting and newly generated CD4+ T-cell responses to SARS-CoV-2 were both impaired in patients with cancer (Salomé and Horowitz, 2021). A multicenter study (Sereno et al., 2021) suggested that prolonged granulocyte-colony stimulating factor (G-CSF) treatment was associated with a worse outcome in cancer patients with neutropenia and COVID-19. Administration of convalescent plasma to patients with hematologic cancers and COVID-19 might be associated with a survival benefit (Thompson et al., 2021).

Cancer patients might be more susceptible to COVID-19 (Sinha and Kundu, 2021; Wang et al., 2021), and there is a great need to carefully protect and monitor cancer patients as part of the strategy to control the COVID-19 pandemic. A bioinformatics analysis (Huang et al., 2020b) suggested that patients with cancers of the respiratory, digestive, or urinary tracts might be particularly more vulnerable to SARS-CoV-2 infection. However, a Mendelian randomization study (Li et al., 2022) reported that cancers might have no causal effect on increasing COVID-19 risk. A real-life setting report (Lamtai et al., 2022) recommended that cancer patients should take the COVID-19 vaccines, which might reduce the mortality rate in cancer patients with COVID-19 (Safari et al., 2022), and should follow their vaccination schemes under the supervision of their treating physicians. Vitamin D supplementation and sun exposure may also be helpful for cancer patients during the COVID-19 pandemic (Vieth, 2022). Notably, the COVID-19 pandemic may significantly delay the care for cancer patients (Nagar and Formenti, 2020; Hochster, 2021).

Cancers seriously affect survival and quality of life (Perez-Cruz et al., 2018; Herrmann, 2020; Huang et al., 2022a,b). For the management of cancers, comprehensive patient-centered care is vital (Huang et al., 2018a, 2019a,b, 2021d). Chemotherapy and/or radiation-therapy are the major treatment modalities for patients with advanced or metastatic cancers that are not resectable and for those having undergone resection (Huang et al., 2018b, 2020c, 2021e; Romitan et al., 2020); many of these therapies are cyclical, so in order to achieve the maximal effect of treatment, the concept of continuous care is proposed. In recent years, continuous care is mainly used for chronic diseases, especially after surgical or other interventional procedures (Huang and Shi, 2022; Shi et al., 2022; Huang et al., 2022a,b,c). Continuous care at home and abroad has not yet clearly required care personnel to receive special training, and most of the caring staff are clinical nurses (Kuderer et al., 2020; Poortmans et al., 2020; Bodschwinna et al., 2022; Park et al., 2022). At present, many researches have been carried out on the continuity of nursing, with promising findings revealed. Kirstine et al. (Sibilitz et al., 2013; Ding et al., 2022) conducted a 2-year continuous care intervention for patients after their discharge from hospital, which improved the patients’ self-homecare skills in various settings. Shimada et al. (Baugh et al., 2022) found that continuous care could significantly improve the mood of patients with malignancy, enhance their tolerance to radiation-therapy and chemotherapy, and reduce the incidence of complications. However, the use of continuous care for patients with cancer during the COVID-19 pandemic had been rarely explored.

This study investigated the needs of continuous care for patients with cancer at Ruijin Hospital, Shanghai Jiao Tong University School of Medicine, during the recent omicron wave of COVID-19 in Shanghai, through comprehensive interviews with cancer patients and their caregivers (Belgacem et al., 2013), and explored the factors influencing compliance to continuous care, which provided important reference for the future application of the Internet-based digital healthcare platform (e.g., *via* the WeChat App).

## Materials and methods

### Study design

**Figure 1:**
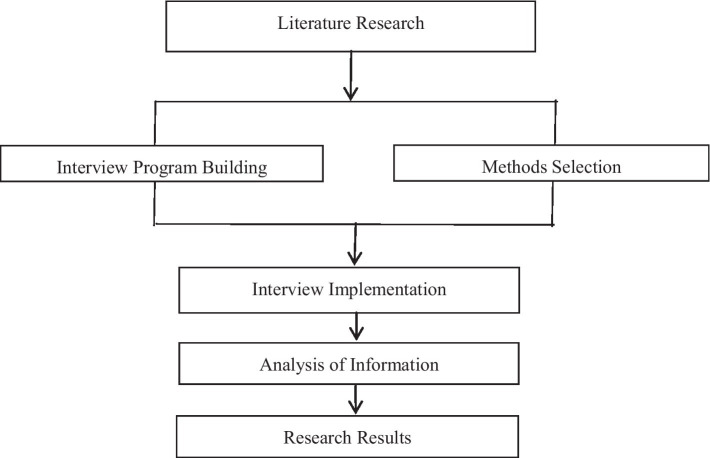
Research roadmap.

A qualitative study (Hwang et al., 2004; Lopez et al., 2019) using the phenomenological approach was applied to investigate the continuous care needs, to clarify the factors affecting the quality of homecare, and to provide reference for constructing a homecare system for patients with cancer receiving chemotherapy during the recent omicron wave of COVID-19 in Shanghai (Figure 1). This approach is an inductive and descriptive method that facilitates understanding the human complexity experience (Hwang et al., 2004) and has been widely used in the field of nursing in recent years (Jiang et al., 2022). This study was approved by the Ethics Committee of Ruijin Hospital. All participants were informed of the purpose of the study in detail by the researchers and that all conversations would be recorded. Participants were could withdraw from the study at any time. Each participant read the informed consent carefully and signed a Consent Statement prior to the start of the interview.

### Participants

Consecutively hospitalized patients with cancer who received chemotherapy from March to May 2022 at Ruijin Hospital, a top tertiary referral hospital in Shanghai, together with some of their homecare-givers, caring oncologists, and oncology nurses, were enrolled for in-depth face-to-face or telephone-based interviews.

Inclusion criteria of patients included: (1) ≥18 years of age; (2) completed ≥1 course of chemotherapy for cancer; (3) without cognitive dysfunction; and (4) able to communicate effectively. The exclusion criteria were as follows: (1) With functional and/or organic mental disorders; (2) with language communication impairments; (3) with suicidal tendencies; and (4) refused to participate in the study.

Inclusion criteria of oncologists and nurses were: (1) Directly provided treatment or nursing care to the enrolled patients and (2) engagement in clinical cancer treatment or care for more than 5 years.

Primary caregivers for interview were randomly selected family members of the patients with cancer who directly participated in the care of the patients and who were 18 to 65 years of age without language communication impairments.

The principles of data saturation were followed (Dueck et al., 2015; Mattsson et al., 2018), and 50 patients (Ps), 4 oncologists (Os), 4 nurses (Ns), and 4 primary caregivers (Cs) were eligible and completed the interview (Tables 1–3).

**Table 1:** Patient and tumor characteristics (*n* = 50).

Variables	Categories	*N* (%) mean ± SD
Gender	Male	30 (60)
	Female	20 (40)
Age (years)	Youth (18–44)	7 (14)
	Middle-aged (45–59)	18 (36)
	Older adult (60+)	25 (50)
Occupation	On-the-job	7 (14)
	Retired	43 (86)
Education	Primary school	7 (14)
	Middle school	18 (36)
	High school	11 (22)
	Junior college	9 (18)
	Undergraduate	4 (8)
	Postgraduate	1 (2)
Marital status	Unmarried	3 (6)
	Married	47 (94)
Drinking history	Yes	20 (40)
Smoking history	Yes	22 (44)
Tumor type	Colon cancer	17 (34)
	Rectal cancer	15 (30)
	Stomach cancer	7 (14)
	Bronchus and lung cancer	2 (4)
	Breast cancer	1 (2)
	Other cancers	5 (10)
ECOG performance status	0	2 (4)
	1	36 (72)
	2	10 (20)
	3	2 (4)
	4	0 (0)
	5	0 (0)
TNM	I	26 (52)
	II	18 (36)
	III	5 (10)
	IV	1 (2)
Tumor histology	Adenocarcinoma	35 (70)
	Ductal and lobular cancer	1 (2)
	Squamous cell cancer	3 (6)
	Cystic, mucinous, and serous cancer	3 (6)
	Others	5 (10)
	Unspecified	3 (6)
Resectional surgery	Yes	38 (76)
History of hypertension	Yes	20 (40)
History of diabetes	Yes	10 (20)
History of cataract	Yes	3 (6)
History of coronary heart disease	Yes	3 (6)
History of arrhythmia	Yes	4 (8)
History of cirrhosis	Yes	3 (6)
History of major abdominal surgery	Yes	39 (78)
History of intake of antihypertensive drug	Yes	18 (36)
History of intake of hypoglycemic drug	Yes	11 (22)
History of intake of anticoagulant	Yes	4 (8)
History of intake of analgesic	Yes	7 (14)
History of intake of sedative	Yes	3 (6)
History of intake of psychotropic drug	Yes	4 (8)
History of intake of immunosuppressive drug	Yes	3 (6)
Height (cm)	-	167 ± 8
Weight on first admission (kg)	-	62 ± 11
Body mass index on first admission (kg/m^2^)	-	22.4 ± 3
Weight loss on first admission	Yes	10 (20)
Basic diet on first admission	Common diet	27 (54)
	Soft or semi-fluid diet	6 (14)
	Others	17 (34)
Low-salt diet on first admission	Yes	16 (32)
Diabetes diet on first admission	Yes	10 (20)
Risk of malnutrition on first admission	Yes	9 (18)
Risk of falling on first admission	Yes	4 (8)
Number of interviews	Face-to-face interviews	35 (70)
	Telephone-based interviews	15 (30)

SD, standard deviation; ECOG, Eastern Cooperative Oncology Group; TNM, tumor-node-metastasis.

**Table 2:** Oncologist (O) and nurse (N) information (*n* = 8).

Participant ID	Gender	Age (years)	Job title	Education level	Experience in oncology treatment or nursing (years)
O1	Male	50	Chief physician	PhD	25
O2	Male	32	Associate researcher	PhD	15
O3	Male	32	Attending physician	PhD	6
O4	Female	31	Attending physician	PhD	5
N1	Female	39	Deputy chief nurse	Master’s degree	20
N2	Female	40	Nurse-in-charge	Undergraduate	21
N3	Female	24	Nurse	Undergraduate	6
N4	Female	33	Nurse-in-charge	Undergraduate	13

**Table 3:** Patient caregiver (C) information (*n* = 4).

Participant no.	Gender	Age (years)	Occupation	Education	Relationship with patient	Related patient
C1	Male	36	On-the-job	Junior college	Son	P12
C2	Male	56	On-the-job	High school	Husband	P15
C3	Female	50	On-the-job	High school	Wife	P18
C4	Male	56	On-the-job	High school	Father	P20

### Data collection

This qualitative study used the phenomenological method (Sun et al., 2020), with face-to-face or telephone-based semi-structured interviews conducted. The audio recording was made prior to the interview, which informed the purposes, significances, and time required. To protect the interviewees’ privacy, their names were masked with numbers (e.g., “Patient 1″) instead of participant names used, while other identifying information was removed. Before the interview, the interviewees were told about the purpose and significance of the study, asked to honestly answer the questions, and acknowledged for the strong support. After the participants learned about the role and license of the recording, interviews began and were recorded throughout the course. Notes were recorded carefully, including those on the interviewees’ facial expressions. Each interview lasted about 30–40 min. After the interview, the recording was repeatedly listened and transcribed verbatim into text materials. Guidance and outlines for the semi-structured interviews on continuous needs for different interviewee populations are shown in Table 4.

**Table 4:** Interview outline.

**For patients**
1. What do you think of the continuous care given when receiving chemotherapy during COVID-19?
2. How do you implement continuous care after you are discharged from the hospital during COVID-19?
3. What factors do you think affect your quality of life when receiving treatment during COVID-19?
4. What kind of guidance do you think is needed for the follow-up after discharge from the current hospital during COVID-19?
5. Do you have any suggestions for continuous care when receiving treatment during COVID-19?
**For family members of patients**
1. What are your problems and needs during your stay at home after the COVID-19 outbreak?
2. What do you think is needed to help patients get through the treatment interval during COVID-19?
**For caring oncologists and oncology nurses**
1. What do you think about continuous care for patients receiving chemotherapy during COVID-19?
2. Do you think there is any difficulty in implementing the Internet-based care after discharge during COVID-19? What aspects does it include?
3. Do you think there is a need to train medical staff on continuous care knowledge for cancer patients during COVID-19? Why?
4. What role do you think continuous care plays in tumor treatment during COVID-19?
5. What do you know about the continuity of care in the current scenario during COVID-19?

### Data analysis

The qualitative data were analyzed using the Colaizzi method (Kim and Yoon, 2022). Qualitative thematic analysis was used to code interview transcripts and create categories of continuous care needs (Liu et al., 2020). Data collection was carried out through post-appointment interviews with the interviewees. The audio recordings were transcribed within 24 h after each interview; original data were read repeatedly; interview situations were recalled; site notes were recorded; and interview materials were marked, kept, and backed up in detail. The content analysis method was used to summarize and refine the theme, and the specific steps were as follows:

Stage 1: All interviews were recorded using audio equipment and transcribed within 24 h.
Stage 2: Important statements pertaining to continuous care needs and directly related to the viewpoints and experiences of patients, homecare-givers, nurses, and physicians during the COVID-19 pandemic were re-read, underlined, extracted, and numbered manually.
Stage 3: Meanings from all significant statements were summarized. During this process, the composition of meaning was reviewed by two PhDs with extensive experience in qualitative research.
Stage 4: The summarized meanings were classified into theme clusters. During this process, the researchers compared the theme clusters to the original data to determine agreement and repeated these processes several times.
Stage 5: Six subthemes were identified in accordance with the study aims, and exhaustive descriptions were developed.
Stage 6: Similar subthemes were organized into larger clusters, and three main themes were obtained.
Stage 7: Essential structures were returned to the participants to verify whether the content was consistent with their perceptions and experiences during the COVID-19 pandemic.

In case of disagreement, a consensus was reached by discussion. All participants consented to be contacted once more and supplied their phone numbers to the researchers.

### Methods of rigor

Four criteria defined by Lincoln and Guba were used to ensure the methodological rigor: Credibility, confirmability, dependability, and transferability (Neubauer et al., 2019). To improve the credibility of the data, the final results were sent to all participants for confirmation and approval. Confirmability was ensured through a clear description of the study context, sampling, and the process for data collection and analysis. As for dependability, two external experts experienced in qualitative research reviewed the decision cues as well as the study’s findings and conclusions. Regarding transferability, the interviewees we recruited varied in many clinical and pathologic factors including age, gender, occupation, level of education, work status, and tumor type and stage (Table 1). This report was guided by the Comprehensive Standard for Reporting Qualitative Research (COREQ; Afshari et al., 2022).

## Results

### Baseline characteristics

Participant characteristics are detailed in Tables 1–3. A total of 50 patients [female, 20 (40%); male, 30 (60%)] were interviewed in this study. The participants were aged 24–75 years, with an average age of 49 years. In terms of education, 7 (14%) were primary school-educated, 18 (36%) were middle school-educated, 11 (22%) had completed high school, 9 (18%) were junior college graduates, 4 (8%) were undergraduates, and 1 (2%) was a postgraduate. In terms of work status, 43 (86%) were retired or unemployed and 7 (14%) were in service.

The interviewers had been trained to conduct unified professional interviews using open-ended questions on the subject. The interview time ranged from 30 to 40 min, with a mean (± standard deviation) of 33.9 (±3.6) min. Data collection and analysis happened simultaneously; after each interview, interview excerpts could be easily extracted from the transcripts, and verbatim reading excerpts were repeatedly conducted with written records made (Hwang et al., 2004). Three thematic categories emerged from the analysis of the interview data: Disease management needs during COVID-19, medical needs during COVID-19, and spiritual needs during COVID-19 (Table 5).

**Table 5:** Themes and subthemes during COVID-19.

Theme	Sub-theme
1 Disease management needs	1.1 Needs for knowledge of managing adverse events associated with chemotherapy
	1.2 Needs for treatment-related information
2 Medical needs	2.1 Needs for mobile healthcare
	2.2 Needs for medical resources
3 Spiritual needs	3.1 Demands for psychological counseling and interventions
	3.2 Needs for spiritual care

### Theme 1: Disease management needs during COVID-19

#### Needs for knowledge of managing adverse events associated with chemotherapy

With COVID-19 being ongoing, factors such as pandemic panic (Rodriguez-Almagro et al., 2021), inconvenient medical treatment, and worry about hospital cross-infection might reduce disease management for cancer patients. Patients receiving chemotherapy might experience varying degrees of adverse events; if the patients did not understand these events and/or did not have timely access to relevant care, it might do harm to health and lead to psychological concerns such as anxiety, which might influence the effect of the treatment (Efe Erturk and Tasci, 2021). In this study, 44 patients (87.5%) and all the 4 interviewed homecare-givers (Pozet et al., 2016) said that they could not manage symptoms well during the recent omicron wave.

*"After the last hospitalization, the omicron wave broke out. Now there have been a lot of rashes all over my body. I wanted to ask my doctor if there was a good way to relieve the symptoms, but I have not been able to see the doctor in time because of the outbreak of COVID-19.” (P2)*

*"On TV, I saw that people always lost their hair when they received chemotherapy. Why don’t I lose my hair? Is it because chemotherapy is useless for me, or is it because the epidemic did not allow me to receive chemotherapy in time?” (P15)*

*"Why was my gastrointestinal response so great to this chemotherapy after the outbreak compared to the previous chemotherapy?” (P17)*

*"During the outbreak, I felt weak after chemotherapy, and I did not know how to relieve the weakness. How to find a doctor for help?” (P30)*

*"During the outbreak period, some hospitals were used as designated hospitals for COVID-19 patients, and some cancer patients could not be timely informed or easily hospitalized.” (O1)*

*"We need to strengthen education during patient hospitalization. It was not convenient to go out of home during the outbreak, and some patients really did not know how to effectively care for themselves.” (N2)*

#### Needs for treatment-related information

Chemotherapy is a relatively long process, and blood examinations before each chemotherapy session and efficacy evaluations after receiving medication are needed. Patients expressed most concern about not being able to go to the hospital for blood review and evaluation in time due to the outbreak. Thirty-eight patients (62.5%) reported that they wanted more information regarding their conditions and treatment.

*"After chemotherapy, I hope my doctor can tell me what to do at this stage, especially because it is not convenient to see a doctor during the outbreak. I hope my doctor can tell me what I need to pay attention to.” (P1)*

*"Before the outbreak, the doctor gave me a lot of liver protection medicine, and I was also taking it seriously. Why is the liver function still not good with another cycle of chemotherapy again during the outbreak?” (P4)*

*"The gastrointestinal reaction during the days when I am at home after each chemotherapy is more severe than when I receive chemotherapy in hospital, especially when it is more difficult to go to the hospital during the outbreak. Can I make a phone call to require medical visits after the chemotherapy?” (P15)*

*"Our ward used to be an oncology ward. After leaving the hospital, patients used to call us to inquire when to come to the hospital again. It helped them by contacting us in time. Due to the COVID-19 outbreak, our hospital has been transformed into a designated COVID-19-treating hospital, and it is very inconvenient for patients to contact us for relevant matters.” (O4)*

### Theme 2: Medical needs during COVID-19

#### Needs for mobile healthcare

With continuous developments in mobile communication technology and the popularization of smartphones, traditional modes of medical care are changing, and the utilization rate of mobile medical care is increasing. Due to the availability of scientific information online, patients are able to access scientific knowledge even after discharge from the hospital, laying a solid foundation for improving the quality of continuous care after discharge (Hui et al., 2016). Thirty-eight patients (62.5%) in the survey said they needed mobile health care during the COVID-19 pandemic, as access to hospitals was difficult.

*"This outbreak has led to great difficulties for us patients who go to the hospital for chemotherapy regularly. Most importantly, we cannot find a doctor for consultation; we hope to have an online WeChat group where questions can be answered in time.” (P12)*

*"If I feel sick after discharge, I will endure it, because I have to issue a negative nucleic acid report within 24 hours to go to the hospital. I really hope I can communicate with my attending doctor on my mobile phone, and then get door-to-door delivery service.” (P15)*

*"I now live in a nursing home, and I am not allowed to go out at will because of the outbreak. I have survived any discomfort after chemotherapy and discharge. Can the hospital have a special contact platform for nursing knowledge consultation, or even provide door-to-door medical services when necessary?” (P16)*

*"Cancer patients are older on average, have less physical strength and poorer performance status after receiving chemotherapy, and do not know how to deal with adverse events; they can only remember a certain part of repeated health education after discharge from hospital. Patients call our mobile phones during the outbreak; as we sometimes work in cabins, it is not possible to answer or get back to them immediately. Patients need mobile electronic devices where they can receive relevant digital content remotely and regularly.” (N2)*

#### Needs for medical resources

The recent omicron wave of COVID-19 in Shanghai from March 2022 posed a challenge to medical and healthcare resources. Some general hospitals in Shanghai were transformed into hospitals designated to manage COVID-19 patients. Due to this transformation of hospitals, some of the original specialized wards could not manage cancer patients normally. Thirty-eight patients (62.5%) said that it might be difficult to be hospitalized during the outbreak.

*"Sometimes if you want to go to the hospital for medical care, you have to do nucleic acid testing almost a day in advance, which is however unbearable for your body.” (P6)*

*"During the COVID-19 period, some hospitals have become designated hospitals for managing COVID-19 patients. There are few beds for us. It is difficult to find a hospital for chemotherapy, which may delay my chemotherapy.” (P12)*

*"My platelet level is a little low. When I was discharged from the hospital before the outbreak, the doctor prescribed me medicine to boost platelet count. Due to the outbreak, the hospital near my house was transformed into a designated hospital to treat COVID-19 patients, which was inconvenient for cancer patients like me to access necessary care.” (P15)*

*"I am an ostomy patient; I think my biggest worry during treatment is stomy care: I cannot change the pocket, and I need to go to the stomy clinic for help. The difficulties have increased due to the outbreak. I hope professional nurses can come to my home to replace the pocket.” (P18)*

*"I went to the hospital every week to maintain the PICC catheter before the outbreak, and I had bone metastasis and my legs was very painful. I did not want to bother myself every week to go to the hospital during the outbreak.” (P20)*

*"It was troublesome during the outbreak to go to the hospital to maintain my mother's PICC. Two weeks later, I found that the puncture point was a little red, and the doctor said that it was slightly infected.” (C20)*

*"In order to prevent the situation of myelosuppression after discharge, we will give the patient the corresponding cell count-lifting injection. If this could be completed in the community hospital, patients can save themselves from waiting for long in the hospital registration queue.” (O3)*

*"It is suggested that some basic nursing operations for cancer patients can be done in community hospitals.” (N4)*

### Theme 3: Spiritual needs during COVID-19

#### Demands for psychological counseling and interventions

Due to the outbreak of COVID-19, cancer patients might experience fear, anxiety, paranoia, depression, and indifference (Hijazi et al., 2022). The process of chemotherapy is not all smooth; factors such as the effect of medicine, disease progression, and available economic resources for continued treatment will affect the mood of the patients at any time. Patients commonly lack a feeling of security and need communication, encouragement, and reassurance that medical care can be delivered to them. In the present study, 40 (80%) patients reported that they wanted psychological advice on healthcare and living during the outbreak.

*"Cancer is not a general disease, and I am under great psychological pressure, especially during the outbreak; I worry about my condition and infection complication, and it will be very uncomfortable.” (P5)*

*"I hope that the hospital has a department for psychological counseling, which can provide counseling for patients and their families and improve their psychological adaptability. Many people diagnosed with cancer are very scared, and they cannot cooperate with the doctor properly; they cannot cope psychologically. Both patients and their families should equip themselves with relevant knowledge.” (P8)*

*"The COVID-19 has lasted so long, and the hospital waiting cycle has grown. She (P12) becomes very anxious at home and always thinks carelessly. Besides, with ascites, her mood is much more depressed, and I don't know how to comfort her.” (C1)*

*"One of our old patients was infected this time, and he was very anxious. At present, he shows no symptoms and has been admitted to the designated hospital. I'm rather worried that our cancer patients are emotionally unstable during the outbreak.” (O1)*

#### Needs for spiritual care

Spiritual care is a process wherein caregivers help maintain patients’ spiritual comfort by accompanying them, listening to them, and showing empathy after assessing their spiritual distress and needs (Altintas et al., 2018; Pollock et al., 2020). Some patients reported that due to the outbreak, the residential district was locked down and that they could not access the company and care of their family members.

*"Due to the outbreak, my daughter's residential quarter has been sealed off, so she can't come to my home to accompany and take care of me, leaving only me and my wife behind. Ah (sigh)." (P7)*

*"Since I was sick, I have been optimistic in front of my family, but every time in the dead of night, I secretly wipe my tears alone. This became especially apparent during the outbreak, which was almost uncontrollable. I am afraid that my husband and children are worried." (P15)*

*"I'm in my thirties and unmarried. So not only did I not let my father have a grandson, but I also asked him in his 50s to take care of my food and clothing. Thinking of this, I hate myself. The outbreak has increased the inability feeling” (P20)*

## Discussion

During the outbreak of the recent COVID-19 wave, because SARS-CoV-2 was highly infectious and difficult to control, population quarantine and control measures were implemented (Huang et al., 2020a; Huang, 2022). Especially for cancer patients, the pandemic caused issues pertaining to the supply of drugs (Zhang et al., 2016) and access to medical care, interfered with regular examinations and management, and led to the fear of being infected, significantly affecting the physical and mental health of the patients. Continuous care (Huang et al., 2022c) refers to medical caregivers helping patients transition from an acute to a subacute phase of the disease management in a timely and effective manner, and to patients enjoying relevant care at home after discharge (Bradt et al., 2016). Kim and Yoon (2022) suggested that patients’ needs are related to support for treatment-related somatic symptoms (e.g., fatigue, pain, nausea, and vomiting), emotional problems (e.g., fear of relapse, anxiety, and depression), and social problems (e.g., lack of support from family, society, and caregivers) (Zhang et al., 2016; Halemani et al., 2022). At present, there are few researches on continuity of cancer care during the COVID-19 pandemic. This study, by examining the experiences of cancer patients, caregivers (Girgis et al., 2013), and medical staff regarding the demand for continuity of care, aimed to identify place for improvement to help cancer patients correctly understand their physical and mental conditions while maintaining a positive attitude in the face of disease, enhance their confidence to overcome the disease, and alleviate negative emotions during the COVID-19 pandemic (Pinato et al., 2017). This study identified three themes and six subthemes related to the needs of continuous homecare for cancer patients including their medical and spiritual needs, with the joint assessment by medical staff and homecare-givers.

As a place for the diagnosis and treatment of disease, hospitals are the most direct information source for patients. Doctors’, nurses’, and other professionals’ visits and even telephone follow-ups are conducive to meeting the information needs of patients. Most patients and their families in this study reported the adverse drug reactions experienced by them and expressed a hope to have ways to understand the specific countermeasures during the COVID-19 outbreak. Lestari et al. (2021) found that 84% of patients in their study needed effective information support, emotional support, and decision support from medical professionals. At present, many researchers worldwide are studying the use of mobile technology, electronic media, and network information for health education to provide continuity of care (Bargetzi et al., 2021). With the rapid pace of development of mobile technology, the use of these resources is likely to facilitate solving the lack of professional personnel and to promote the implementation of home-based nursing for continuity of nursing intervention. Providing support for the continuation of care for cancer patients through mobile healthcare, especially for older cancer patients, has also been suggested. Telemedicine is one of the most important technological innovations of the late twentieth century which serves as a gateway to modern healthcare. The goal of telemedicine was to improve the quality of care, enhance patient safety, and provide rapid access to healthcare by overcoming geographical barriers. The use of telephones, cellphones, text messages, and communication technologies as part of distance nursing is one form of telemedicine (Neubauer et al., 2019). “Internet + nursing services” should be used to provide older and disabled cancer patients with chronic disease management, rehabilitation nursing, special nursing, health education, and other issues specific to older cancer patients. By making full use of the Internet technology, the burden of patients seeking medical treatment can be reduced such that they can enjoy high-quality nursing services without leaving their homes, the diversified and multi-level health needs of the people can be accurately identified, and patients can be provided with care at more convenience.

The unprecedented pandemic has caused a considerable psychological impact on the general public, leading to the emergence of various psychological problems, including mood disorders, depression, anxiety, and negative personality traits (Liu et al., 2020). In recent years, domestic and foreign studies have confirmed very high incidence of mood disorders in cancer patients, with the incidence of depression in cancer patients being as high as 20%–50% (Bodschwinna et al., 2022; Park and Lim, 2022). The cancer patients in this study were all in home recuperation during COVID-19 and lacked channels and opportunities to access medical care. Therefore, they were eager to establish an interdependence and trust relationship with the researchers and were willing to share their inner demands. In the interviews, many patients mentioned their emotional problems and expressed a desire to understand how to manage their emotions well. The medical staff also said that they should pay attention to meet the psychological safety of patients, and suggested that emotional problems arising during the treatment should be treated reasonably during the outbreak. Timely psychological care and social support for patients can mobilize the subjective initiative of patients, so that patients can act practically and actively to fight against the disease (Chew et al., 2017). Psychological comfort can reduce the occurrence of bad mood in cancer patients during their stay at home. It is also expected to help patients take initiative to actively cooperate with the treatment, enhance their confidence, and increase their determination to overcome the disease. Older adults with cancer often require supportive care due to comorbidities and/or physical, cognitive, and/or functional impairments (Huang et al., 2022c). This study included 25 patients (50%) aged over 60 years. In the clinic, psychological care for older patients with tumors can not only cater to the patients’ emotional needs, but it can also be a convenient mode to facilitate treatment from the perspective of medical staff. Through psychological care for older cancer patients, listening to their hearts, and effectively communicating with them, the occurrence of depression can be greatly reduced, and psychological care could help encourage them to face tumors with an optimistic and calm mind and give them the confidence to overcome the disease (Karnakis et al., 2016).

The proposed “patient-centered” care is considered as a benchmark for providing quality care services to patients with cancer (Mehta et al., 2009). Effective and high-quality cancer care not only provides anticancer treatment, but also ensures that healthcare providers meet the various continuous care needs of their patients. Continuous care (Dueck et al., 2015; Mattsson et al., 2018; Curry et al., 2021) needs involve dealing with both the tumor- and treatment-related physiological responses, such as pain, fatigue, nausea, and vomiting, and psychological and social complications, such as anxiety and depression. Rapid access to information is also one of the key components of continuous care. To ensure that patients’ needs are met, needs must be first carefully assessed. Recent studies have shown that the effect of cancer care is evaluated using multiple strategies, including quality of life assessment, care satisfaction assessment, and needs assessment (Porras-Segovia et al., 2019). The present study comprehensively explored the continuous care needs of cancer patients by interviewing doctors, nurses, patients, and family members.

This qualitative study has some limitations. Due to time and manpower constraints owing to the pandemic, this study was not carried out at multiple centers. The sample size could be further expanded in future research. Furthermore, future research should combine quantitative methods with longitudinal investigation by conducting long-term follow-up surveys of the patients to identify and improve the continuous care needs for cancer patients receiving chemotherapy, and to provide tailored nursing interventions on related topics according to patients’ needs. Some other independent variables, such as differences in personality and acceptance of interview questions, could not be easily controlled for or explained, and such differences might influence patients’ perceptions of continuity of care (Davies and Wood, 2018).

Cancer patients undergoing chemotherapy experience a long-term treatment process (Huang et al., 2014, 2018b, 2019a; Cha et al., 2021), especially during the COVID-19 outbreak. Addressing patients’ needs, including disease management needs, medical needs, and spiritual needs, will affect their and their caregivers’ quality of life and life satisfaction (Wei et al., 2020; Huang et al., 2020c,d, 2021d,e, 2022b,d; Guo et al., 2021). To meet these challenges, medical staff should conduct related health education for patients while they are still in the hospital, provide efficient and convenient continuous nursing measures, and strengthen caregivers’ initiative to ensure that patients actively participate in their treatment process and improvement of their quality of life. We must also know that this type of care requires informed and planned support and sufficient community education. The healthcare system needs to put self-care and family care among its top priorities. The focus should be on educational and mental support of informal caregivers along with measures that protect them and their relatives from COVID-19. With the rapid development of economy and society, “Internet +” is being widely used in different fields (Stuij et al., 2020), but in the medical field, the application of “Internet +” is still nascent. This study investigates the view of continuous care for patients receiving chemotherapy, which can provide important reference for the construction of an Internet + continuous care program, effectively help patients and families to realize the extension and transition from hospital care to homecare, and optimize health management for cancer patients during COVID-19 (James et al., 2016; Kipfer and Pihet, 2020; Stuij et al., 2020).

## Data availability statement

The datasets presented in this article are not readily available because the original data for this study were used under license and are not publicly available. Requests to access the datasets should be directed to JiZ, zj21283@rjh.com.cn.

## Ethics statement

The studies involving human participants were reviewed and approved by the Ethics Committee of Ruijin Hospital. The patients/participants provided their written informed consent to participate in this study. Written informed consent was obtained from the individual (s) for the publication of any potentially identifiable images or data included in this article.

## Author contributions

JiZ and LH: conception or design, drafting of the manuscript, and statistical analysis. JiZ, CW, LH, and JuZ: acquisition, analysis, or interpretation of data, and critical revision of the manuscript for important intellectual content. LH and JuZ: administrative, technical, or material support. All authors contributed to the article and approved the submitted version.

## Funding

This work was supported by Department of Nursing, Ruijin Hospital, Shanghai Jiao Tong University School of Medicine (grant number, RJHK-2021-25).

## Conflict of interest

The authors declare that the research was conducted in the absence of any commercial or financial relationships that could be construed as a potential conflict of interest.

## Publisher’s note

All claims expressed in this article are solely those of the authors and do not necessarily represent those of their affiliated organizations, or those of the publisher, the editors and the reviewers. Any product that may be evaluated in this article, or claim that may be made by its manufacturer, is not guaranteed or endorsed by the publisher.
